# Supplementary material for: A probe for NIR-II imaging and multimodal analysis of early Alzheimer’s disease by targeting CTGF
Source: Nat Commun. 2024 Jun 12;15:5000. doi: 10.1038/s41467-024-49409-4 (PMC11169542; doi:10.1038/s41467-024-49409-4)
Supplement: Supplementary file 1 — Supplementary Information [file 41467_2024_49409_MOESM1_ESM.pdf]

A probe for NIR-II imaging and multimodal analysis of early Alzheimer's disease by targeting CTGF

Cao Lu[a], Cong Meng[a], Yuying Li[b], Jinling Yuan[a], Xiaojun Ren[a], Liang Gao[a], Dongdong Su[a], Kai Cao[a], Mengchao Cui[b], Qing Yuan[*a], Xueyun Gao[*a]

[a] Center of Excellence for Environmental Safety and Biological Effects, Department of Chemistry, Beijing University of Technology, Beijing 100124, P. R. China.

[b] Key Laboratory of Radiopharmaceuticals, Ministry of Education, College of Chemistry, Beijing Normal University, Beijing 100875, P. R. China

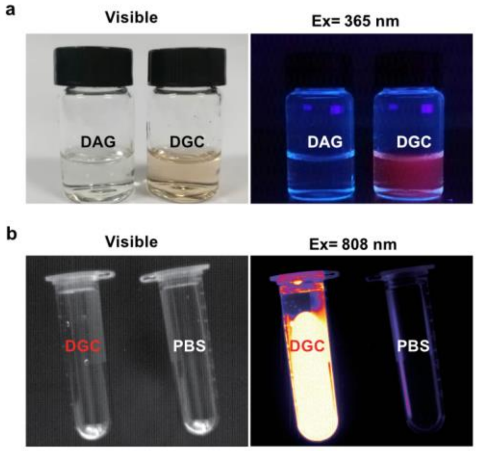

Figure S1. Visible and fluorescence images of DAG and DGC. (a) Digital images of DAG and DGC under sunlight and a 365 nm portable UV light. (b) Digital images of DAG and DGC under sunlight and the excitation intensity of 808 nm radiation.

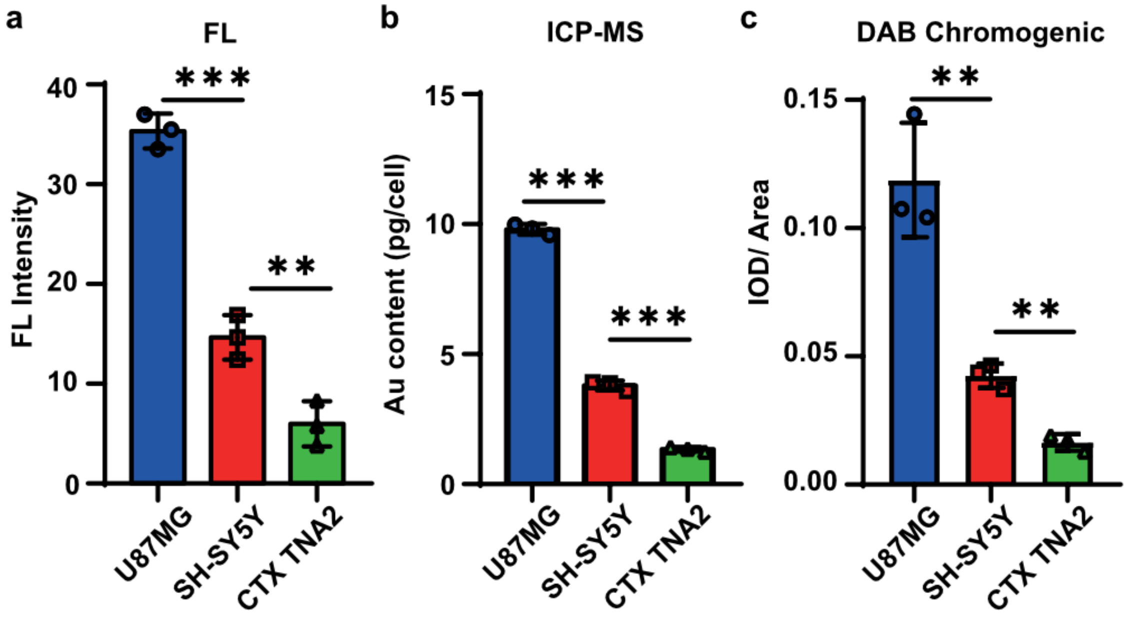

Figure S2. Multimodal quantitative analysis of DGC labelled cell lines. (a) The fluorescence intensity of DGC in Figure 3c analyzed by CLSM software. (b) ICP-MS analysis of DGC (Au) content per cell after incubating DGC with indicated cell lines. (c) The chromogenic imaging intensity in Figure 3c when cell treated by DGC and DAB working solution and analyzed by image pro plus software. Data are presented as mean ± SD from three independent experiments ($n = 3$). **$p<0.01$, ***$p<0.001$, Student's t-test.

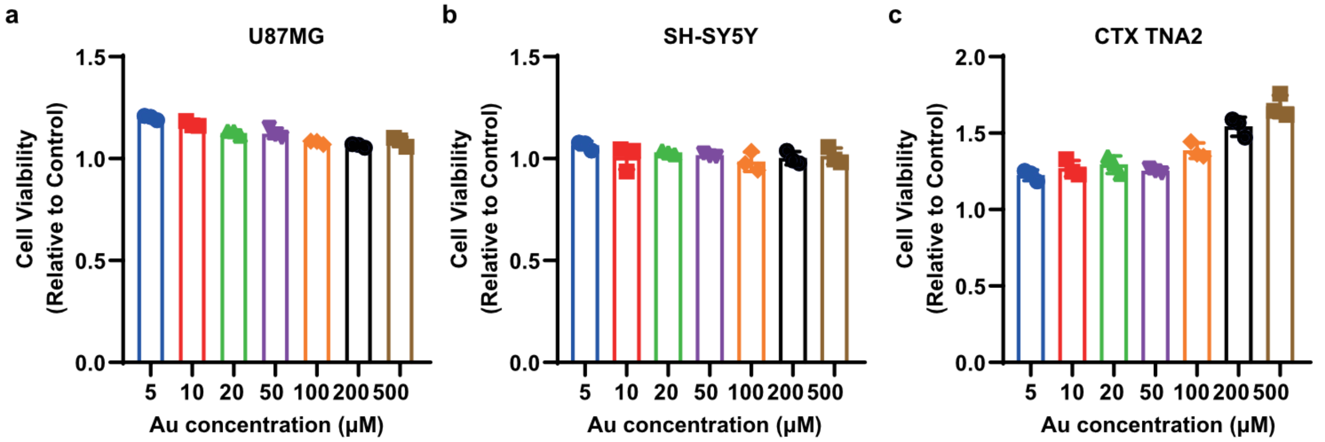

Figure S3. Cytotoxicity of DGC in cell lines. Cytotoxicity of DGC on U87MG cells, SH-SY5Y cells and CTX TNA2 cells was assessed. Data are presented as mean ± SD from three independent experiments (n = 3).

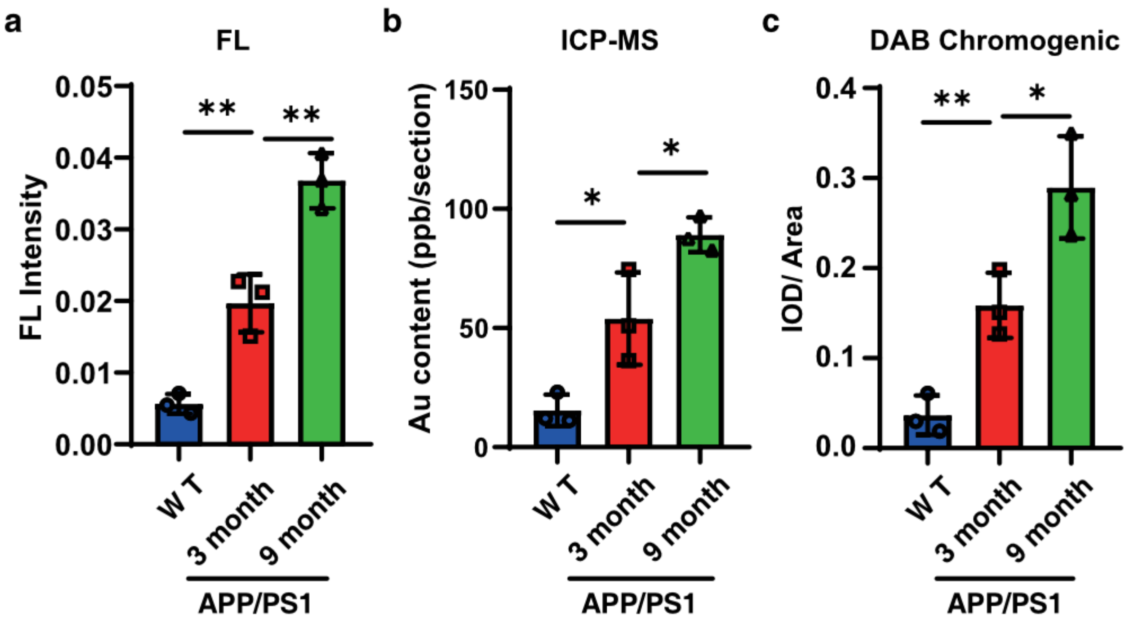

Figure S4. Multimodal quantitative analysis of DGC labelled brain sections of APP/PS1 mice *in vitro*. (a) The fluorescence intensity of DGC in Figure 4a analyzed by CLSM software. (b) ICP-MS analysis of Au content per section after incubating DGC with indicated samples. (c) The chromogenic imaging in Figure 4a when section treated by DGC and DAB working solution and analyzed by image pro plus software. Data are presented as mean ± SD from three independent sections (n = 3). *$p<0.05$, **$p<0.01$, Student's t-test.

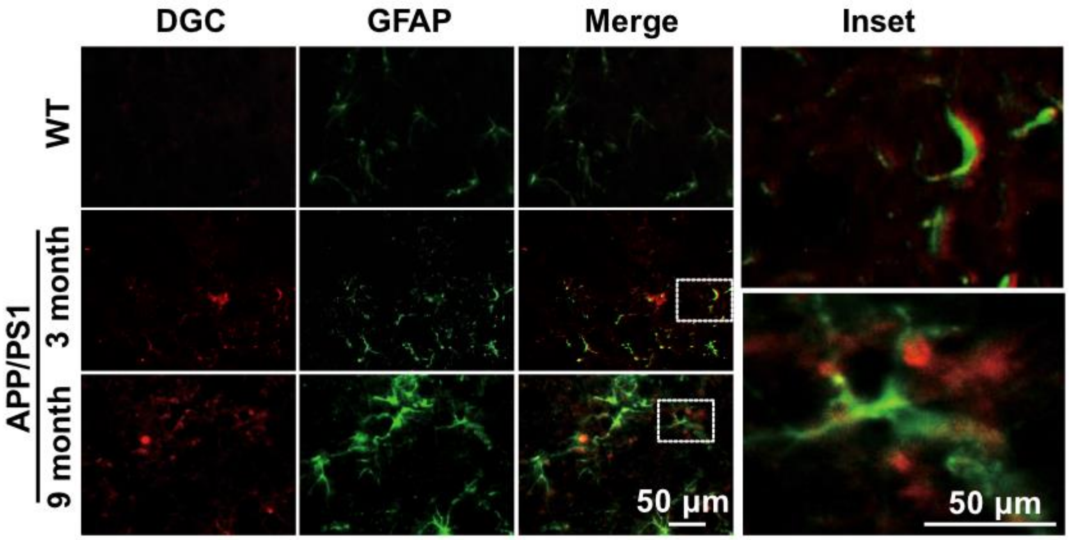

Figure S5. Colocalization of DGC and active astrocytes in brain sections of APP/PS1 mice *in vitro*. Representative images of brain sections from AD mice stained by DGC (red fluorescence) and FITC labeled-GFAP antibody (green fluorescence). The inset pictures show the status of partial colocalization between DGC and GFAP. Scale bar = 50 μm. The experiment was repeated in 3 independent group of brain sections with similar results.

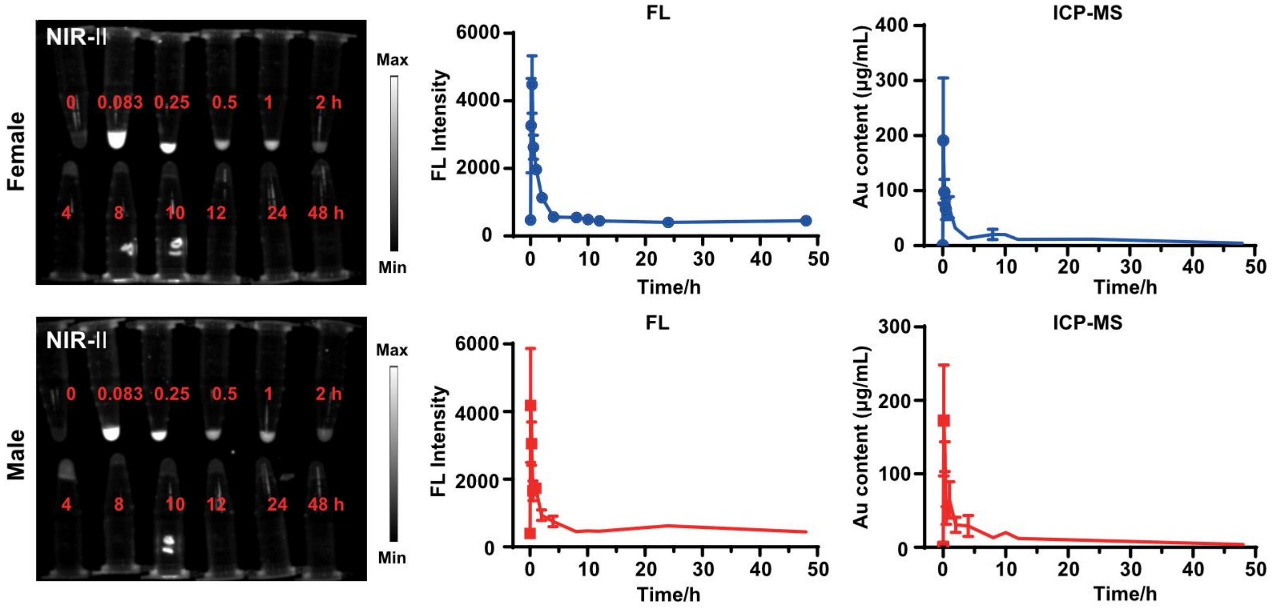

Figure S6. NIR-II fluorescence monitoring and concentration quantification of DGC in serum. The experiment was repeated in 3 independent mice with similar results (male, n = 3; female, n = 3).

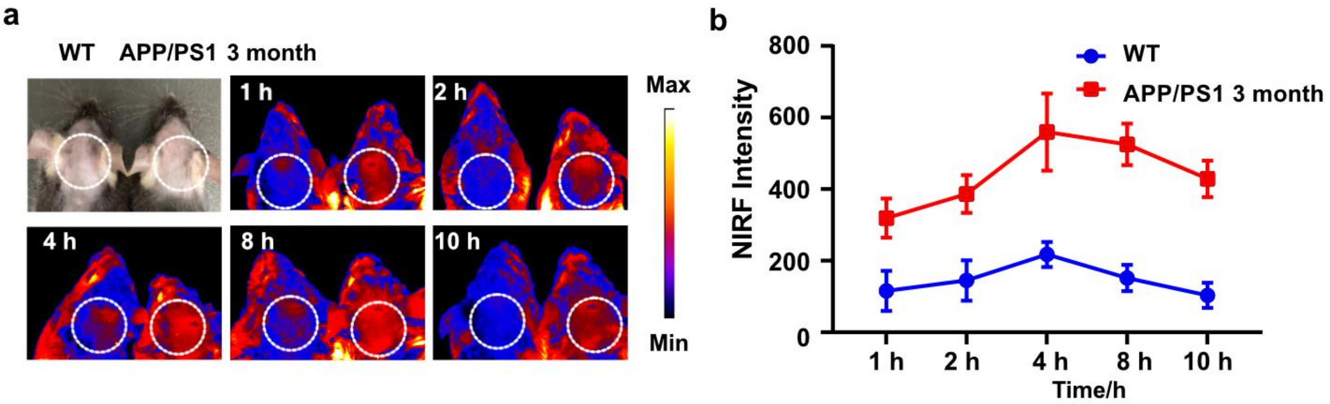

Figure S7. Time-dependent *in vivo* NIR-II imaging for AD mice. (a) Representative NIR-II images from AD mice at serial time points after injection of DGC. The excitation intensity of 808 nm laser. The experiment was repeated in 3 independent pair of mice with similar results. (b) Fluorescence intensity of brain in WT mice and 3-month-old APP/PS1 mice. Data are presented as mean ± SD from three independent mice (n = 3).

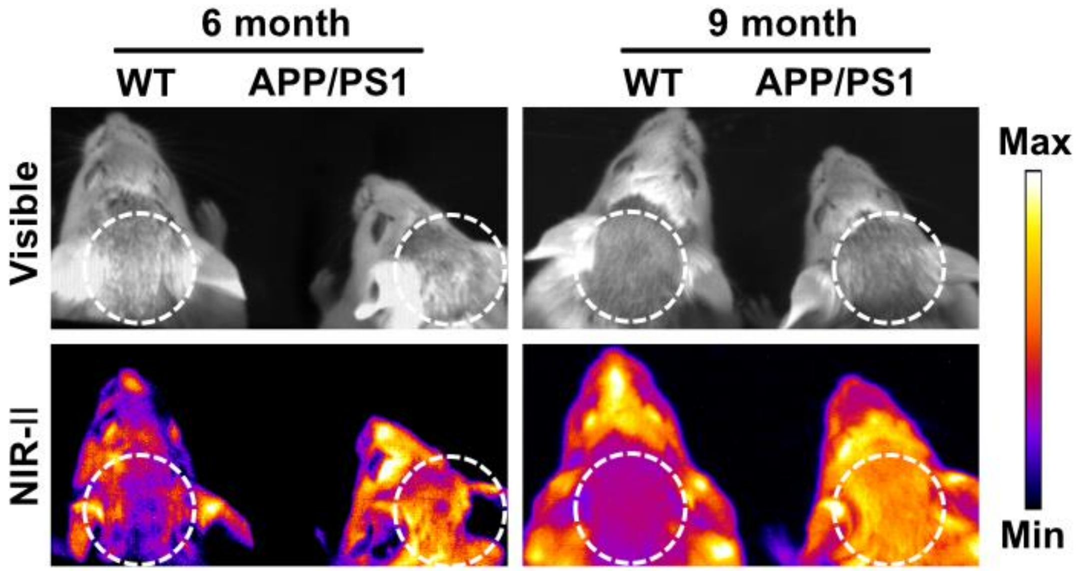

Figure S8. *in vivo* NIR-II imaging of DGC in established AD mice (6- and 9-month-old APP/PS1) and the age-matched WT mice. The experiment was repeated in 3 independent pair of mice with similar results.

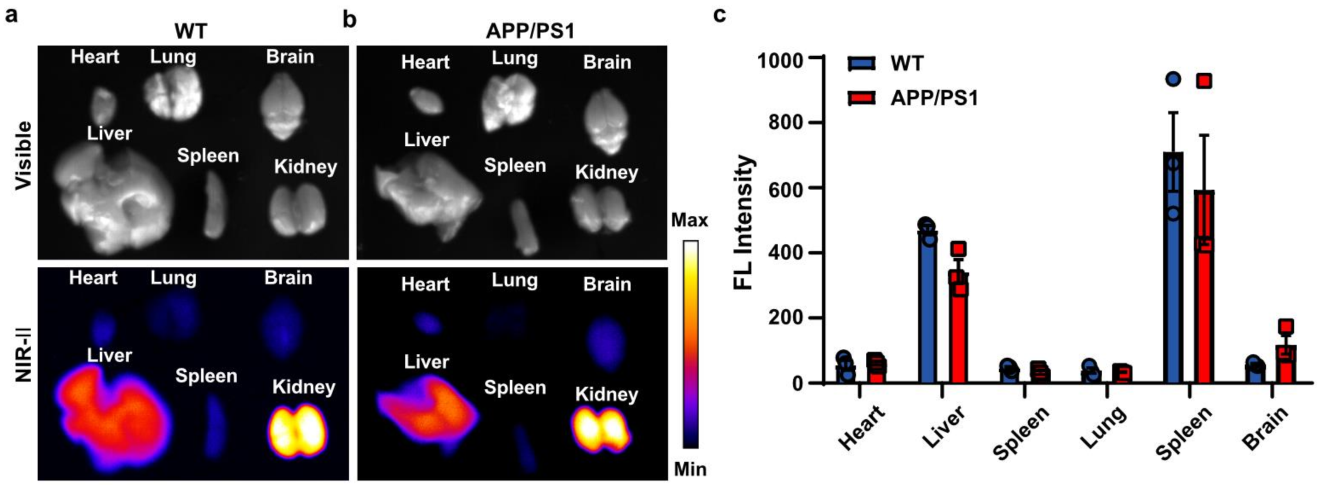

Figure S9. Biodistribution of DGC in WT and APP/PS1 mice detected by NIR-II imaging. Organs dissected from (a) wild type mice and (b) APP/PS1 mice after intravenous injection of DGC for 4 h. The experiment was repeated in 3 independent pair of mice with similar results. (c) The fluorescence intensity of DGC in Organs dissected from WT and APP/PS1 analyzed by Image J software. Data are presented as mean ± SD from three independent mice (n = 3).

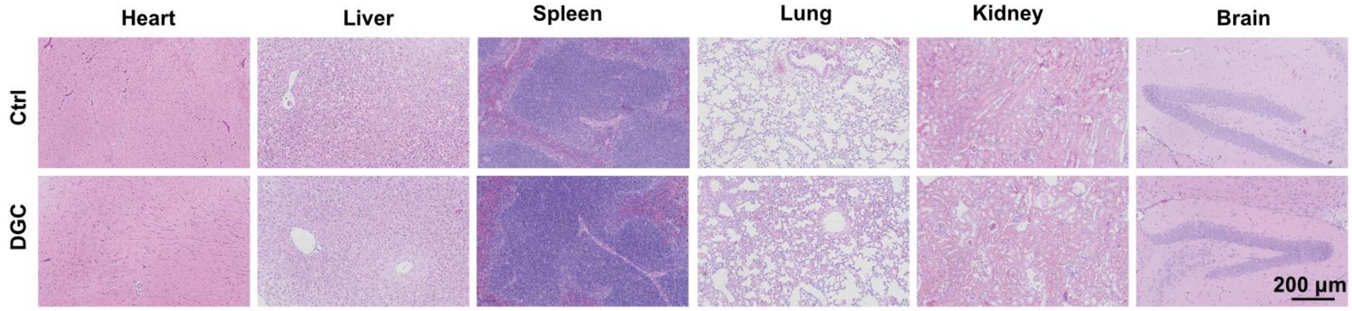

Figure S10. Representative H&E staining of heart, liver, spleen, lung, kidney and brain in DGC injected C57/6J mice (n = 3). The experiment was repeated in 3 independent pair of mice with similar results. Scale bar = 200 μm

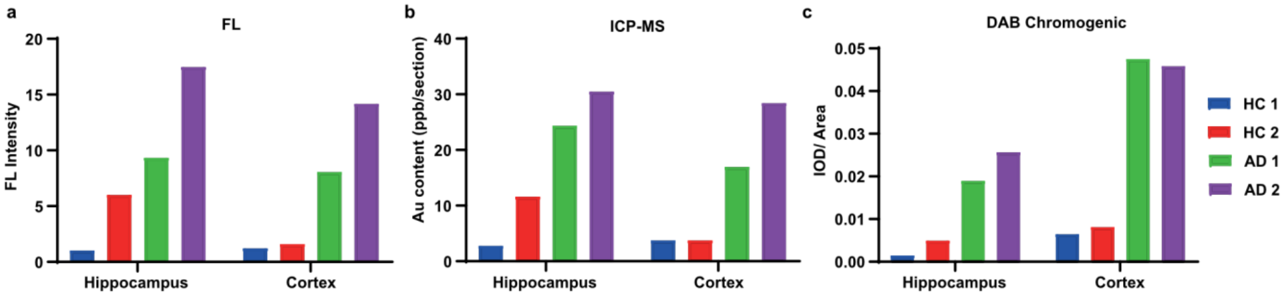

Figure S11. Multimodal quantitative analysis of DGC labelled brain sections of AD patient. (a) The fluorescence intensity of DGC in Figure 7a analyzed by CLSM software. (b) ICP-MS analysis of Au content per section after incubating DGC with indicated samples. (c) The chromogenic imaging in Figure 7c when section treated by DGC and DAB working solution analyzed by image pro plus software.

Table S1. The effective permeability coefficient of DGC

Sample	PAMPA permeability Mean Pe (10^{-6} cm/s)
DGC	2.15 ± 0.44 (n = 3)

Classification Criteria: PAMPA permeability Pe > 1.5 * 10^{-6} cm/s→ high permeability; Pe < 1.5 * 10^{-6} cm/s→ low permeability.

Table S2. Values of parameters of the Pharmacokinetic of DGC in C57/6J mice

Parameter	Unit	Value	
		Female (n=3)	Male (n=3)
AUC(0-t)	h*mg/L	651.00	615.22
AUC(0-∞)	h*mg/L	779.37	735.65
AμMC(0-t)	h*h*mg/L	10136.52	8803.77
AμMC(0-∞)	h*h*mg/L	19964.46	18299.37
MRT(0-t)	h	15.57	14.31
MRT(0-∞)	h	25.62	24.88
t1/2z	h	19.80	21.38
Tmax	h	0.08	0.08
CLz/F	L/h/kg	0.03	0.03
Vz/F	L/kg	0.73	0.84
Cmax	mg/L	191.26	172.69

Table S3. Effect of DGC on hematology of mice

Parameter	Short name	Ctrl results (n = 3)	DGC results (n = 3)	Unit	Normal parameter range
White blood cell count	WBC	7.12 ± 2.72	8.03 ± 1.12	10^9/L	0.80 - 10.60
Neutrophil count	Neu#	1.08 ± 0.6	1.07 ± 0.24	10^9/L	0.23 - 3.60
Lymphocyte count	Lym#	5.74 ± 1.84	6.65 ± 0.78	10^9/L	0.60 - 8.90
Monocytes count	Mon#	0.23 ± 0.26	0.25 ± 0.1	10^9/L	0.04 - 1.40
Eosinophil granulocyte count	Eos#	0.05 ± 0.04	0.04 ± 0.02	10^9/L	0.00 - 0.51
Basophil granulocyte count	Bas#	0.01 ± 0.01	0.02 ± 0.01	10^9/L	0.00 - 0.12
Percentage of neutrophil	Neu%	14.47 ± 3.87	13.2 ± 1.31	%	6.5 - 50.0
Percentage of lymphocyte	Lym%	81.93 ± 5.85	83 ± 2.08	%	40.0 - 92.0
Percentage of monocytes	Mon%	2.7 ± 2.23	3.07 ± 0.84	%	0.9 - 18.0
Percentage of eosinophil granulocyte	Eos%	0.67 ± 0.31	0.47 ± 0.23	%	0.0 - 7.5
Percentage of Basophil granulocyte	Bas%	0.23 ± 0.12	0.27 ± 0.06	%	0.0 - 1.5
Red blood cell count	RBC	10.45 ± 0.27	10.16 ± 0.42	10^{12}/L	6.50 - 11.50
Hemoglobin	HGB	166.67 ± 5.51	161.67 ± 8.02	g/L	110 - 165
Hematocrit	HCT	52 ± 0.87	51.2 ± 1.93	%	35.0 - 55.0
Mean corpuscular volume	MCV	49.77 ± 0.45	50.4 ± 0.17	fL	41.0 - 55.0
Mean hemoglobin content of red blood cells	MCH	15.93 ± 0.31	15.93 ± 0.25	pg	13.0 - 18.0
Mean concentration of red blood cell hemoglobin	MCHC	320 ± 8.19	315.67 ± 5.69	g/L	300 - 360
Coefficient of variation of red blood cell distribution width	RDW-CV	13.7 ± 0.3	13.13 ± 1	%	12.0 - 19.0
Standard deviation of red blood cell distribution width	RDW-SD	28.77 ± 0.95	27.8 ± 2.1	fL	23.0 - 39.0
Platelet count	PLT	1359 ± 102.83	1228.67 ± 179.01	10^9/L	400 - 1600
Mean platelet volume	MPV	5.53 ± 0.15	5.53 ± 0.15	fL	4.0 - 6.2
Width of platelet distribution	PDW	15.47 ± 0.06	15.23 ± 0.25		12.0 - 17.5
Thrombocytopenia	PCT	0.75 ± 0.04	0.68 ± 0.09	%	0.100 - 0.780

Table S4. Effect of DGC on blood Biochemistry of mice

Parameter	Short name	Ctrl results (n = 3)	DGC results (n = 3)	Unit	Normal parameter range
Alanine aminotransferase	ALT	33.87 ± 1.87	32.8 ± 4.26	U/L	10.06-96.47
Aspartate aminotransferase	AST	150.90 ± 44.96	120.2 ± 20.04	U/L	36.31-235.48
Alkaline phosphatase	ALP	206.00 ± 89.66	220.7 ± 27.07	U/L	22.52-474.35
Urea	UREA	11.16 ± 0.92	9.62 ± 1.25	mmol/L	10.81-34.74
Creatinine	CREA	22.87 ± 2.79	22.67 ± 1.98	μmol/L	10.91-85.09

Table S5. The detailed information of brain tissue donors from CBBC

ID Number	Average age	Gender	Parts	The cause of death
HC 1	80.5Y	F	Hippocampal, Cortex	Respiratory failure, heart disease, post-release hypertension
HC 2		M	Hippocampal, Cortex	Respiratory failure, high blood pressure
AD 1		M	Hippocampal, Cortex	AD, atherosclerosis
AD 2		F	Hippocampal, Cortex	AD

Table S6. The detailed information of early-stage AD brain donors from Human Brain Bank

ID Number	Average age	Gender	Pathological diagnosis	Aβ staging (Thal)
Case 1	82.75Y	M	Health control (HC)	0
Case 2		F	Health control (HC)	0
Case 3		M	AD	1
Case 4		M	AD	1

Table S7. Instrumental parameters of LA-ICP-TOFMS for mouse tissue Au imaging

	Parameter
ICP-TOFMS	
RF power	1550W
Nebulizer gas	0.89 L/min
Auxiliary gas	0.8 L/min
Plasma gas	14.0 L/min
Measurement mode	CCT mode
CCT flow	4.5 mL/min
m/z range	14-256
Cone materials	Ni
Notch mass	28, 32, 40, 80
Injector diameter	2 mm
TOF extraction time	46 µs
Laser wavelength	193 nm
Sample introduction system	ARIS
Ablation mode	Spot ablation
Spot size	10 µm
Laser fluency	0.10 J /cm2
Stage movement	2000 µm/s
He flow of inner cell	0.45 L/min
He flow of outer cell	0.15 L/min
Laser repetition rate	200 Hz
