# Supplementary material for: A probe for NIR-II imaging and multimodal analysis of early Alzheimer’s disease by targeting CTGF
Source: Nat Commun. 2024 Jun 12;15:5000. doi: 10.1038/s41467-024-49409-4 (PMC11169542; doi:10.1038/s41467-024-49409-4)
Supplement: Supplementary file 2 — Reporting Summary [file 41467_2024_49409_MOESM2_ESM.pdf]

nature portfolio

Corresponding author(s): Xueyun Gao

Last updated by author(s): May 28, 2024

Reporting Summary

Nature Portfolio wishes to improve the reproducibility of the work that we publish. This form provides structure for consistency and transparency in reporting. For further information on Nature Portfolio policies, see our Editorial Policies and the Editorial Policy Checklist.

Statistics

For all statistical analyses, confirm that the following items are present in the figure legend, table legend, main text, or Methods section.

n/a	Confirmed	
☐	☒	The exact sample size (*n*) for each experimental group/condition, given as a discrete number and unit of measurement
☐	☒	A statement on whether measurements were taken from distinct samples or whether the same sample was measured repeatedly
☐	☒	The statistical test(s) used AND whether they are one- or two-sided<br>*Only common tests should be described solely by name; describe more complex techniques in the Methods section.*
☒	☐	A description of all covariates tested
☒	☐	A description of any assumptions or corrections, such as tests of normality and adjustment for multiple comparisons
☐	☒	A full description of the statistical parameters including central tendency (e.g. means) or other basic estimates (e.g. regression coefficient) AND variation (e.g. standard deviation) or associated estimates of uncertainty (e.g. confidence intervals)
☐	☒	For null hypothesis testing, the test statistic (e.g. *F*, *t*, *r*) with confidence intervals, effect sizes, degrees of freedom and *P* value noted<br>*Give P values as exact values whenever suitable.*
☒	☐	For Bayesian analysis, information on the choice of priors and Markov chain Monte Carlo settings
☒	☐	For hierarchical and complex designs, identification of the appropriate level for tests and full reporting of outcomes
☒	☐	Estimates of effect sizes (e.g. Cohen's *d*, Pearson's *r*), indicating how they were calculated

Our web collection on statistics for biologists contains articles on many of the points above.

Software and code

Policy information about availability of computer code

Data collection	Zetasizer software (version 7.12) was used to collect data of size and zeta potential of nanoprobe, UV Probe Software (version 2.70), FL Solutions (version 4.2) and softMax Pro (version 7.0) was used to collect data of ultraviolet-visible absorption spectrum and fluorescence spectrum: Nls-Elements Viewer Software (version 5,.21.0.14830) was used to collect fluorescence imaging data.
Data analysis	Image J (version 1.8.0) was used to process fluorescence imaging data. GraphPad Prism (version 8.1.244) and Origin 2022 (version 9.90.225) were used for the statistical analysis.

For manuscripts utilizing custom algorithms or software that are central to the research but not yet described in published literature, software must be made available to editors and reviewers. We strongly encourage code deposition in a community repository (e.g. GitHub). See the Nature Portfolio guidelines for submitting code & software for further information.

Data

Policy information about availability of data

All manuscripts must include a data availability statement. This statement should provide the following information, where applicable:

- Accession codes, unique identifiers, or web links for publicly available datasets
- A description of any restrictions on data availability
- For clinical datasets or third party data, please ensure that the statement adheres to our policy

Provide your data availability statement here.

Research involving human participants, their data, or biological material

Policy information about studies with human participants or human data. See also policy information about sex, gender (identity/presentation), and sexual orientation and race, ethnicity and racism.

Reporting on sex and gender	Only postmortem brain tissue slices from human were used in this study, including three male and one female AD patient and two male and two female healthy control.
Reporting on race, ethnicity, or other socially relevant groupings	These samples were obtained from the Chinese Brain Bank Center (CBBC) and Human Brain Bank, Central South University Xiangya School of Medicine based on materials availability, they are all Han Chinese.
Population characteristics	one male AD patient: the cause of death: AD, atherosclerosis<br>one female AD patient: the cause of death: AD<br>one male normal control: the cause of death: Respiratory failure, high blood pressure<br>one female normal control: the cause of death: Respiratory failure, heart disease, post-release hypertension<br>one male AD patient: the Aβ staging (Thal): 1<br>one male AD patient: the Aβ staging (Thal): 1<br>one male health control: the Aβ staging (Thal): 0<br>one female health control: the Aβ staging (Thal): 0
Recruitment	Based on the availability of brain sections from the Chinese Brain Bank Center (CBBC) and Human Brain Bank, Central South University Xiangya School of Medicine based on materials availability.
Ethics oversight	This study was performed ex vivo and was approval by the Ethics Committee of Beijing University of Technology

Note that full information on the approval of the study protocol must also be provided in the manuscript.

Field-specific reporting

Please select the one below that is the best fit for your research. If you are not sure, read the appropriate sections before making your selection.

☒ Life sciences ☐ Behavioural & social sciences ☐ Ecological, evolutionary & environmental sciences

For a reference copy of the document with all sections, see nature.com/documents/nr-reporting-summary-flat.pdf

Life sciences study design

All studies must disclose on these points even when the disclosure is negative.

Sample size	In vivo or vitro experiments were performed in at least three bioindependent samples and which is sufficient for statistical purposes.
Data exclusions	No data was excluded in this study.
Replication	All data came from at least three biological independent replications and presented as mean ± SD.
Randomization	All animals were numbered and randomly allocated into each group by random number table method. For cell experiments, randomization was not applied due to all samples were performed equally.
Blinding	All investigators were blinded to group allocation during data collection and analysis.

Reporting for specific materials, systems and methods

We require information from authors about some types of materials, experimental systems and methods used in many studies. Here, indicate whether each material, system or method listed is relevant to your study. If you are not sure if a list item applies to your research, read the appropriate section before selecting a response.

Materials & experimental systems

n/a	Involved in the study	
☐	☒	Antibodies
☐	☒	Eukaryotic cell lines
☒	☐	Palaeontology and archaeology
☐	☒	Animals and other organisms
☒	☐	Clinical data
☒	☐	Dual use research of concern
☒	☐	Plants

Methods

n/a	Involved in the study	
☒	☐	ChIP-seq
☒	☐	Flow cytometry
☒	☐	MRI-based neuroimaging

Antibodies

Antibodies used

Rabbit anti-CTGF antidoby (Cat#ab6992; Abcam), Neun (Cat#ab177487; Abcam), Rabbit anti-A 17-24 (4G8) antibody (Cat#SIG-39200; BioLegend),Rabbit anti-GFAP Polyclonal antibody (Cat#16825-1-AP; Proteintech), Anti-CD31antibody (CatAF6408; Beyotime), Rabbit anti-Iba 1 antibody (Cat#AF7143; Beyotime), Rabbit Anti-actin (Cat#bs-10966R; Bioss), HRP-labeled Goat AntiRabbit IgG(H+L) (Cat#A0208Beyotime), FITC-labeled Goat Anti-Rabbit IgG (H+L) (Cat#bs-0370R-FITC; Bioss).

Validation

https://www.abcam.com/products/primary-antibodies/ctgf-antibody-ab6992.html
https://www.abcam.com/products/primary-antibodies/neun-antibody-epr12763-neuronal-marker-ab177487.html
https://www.ptglab.com/Products/GFAP-Antibody-16825-1-AP.htm
https://www.biolegend.com/en-gb/products/purified-anti-beta-amyloid-17-24-antibody-11233?GroupID=BLG15648
https://www.beyotime.com/product/af6408.htm
https://www.beyotime.com/product/AF7143.htm
https://www.biossusa.com/products/bs-10966r
https://www.beyotime.com/product/A0208.htm
http://bioss.com.cn/prolook_03.asp?id=AF08169606011161&pro37

Eukaryotic cell lines

Policy information about cell lines and Sex and Gender in Research

Cell line source(s)

Human astrocytoma cell line (U87MG), Neuroblastoma cell line (SH-SY5Y) and normal rat astrocytes line (CTX TNA2) were purchased from the Cancer Institute and Hospital, Chinese Academy of Medical Sciences.

Authentication

The cell lines were authenticated by the manufacturers and supported by multiple publications. In this study, we authentication these cells through CTGF expression detection.

Mycoplasma contamination

The cells were regularly performed with mycoplasma detection and were confirmed negative for mycoplasma contamination.

Commonly misidentified lines
(See ICLAC register)

No commonly misidentified cell lines were used in this study.

Animals and other research organisms

Policy information about studies involving animals; ARRIVE guidelines recommended for reporting animal research, and Sex and Gender in Research

Laboratory animals

The 1-, 2-, 3-, 6- and 9-month-old double transgenic (APP/PS1) AD model mice and the age-matched wild-type C57BL/6J mice were purchased from Beijing HFK Bioscience Co., Ltd.

Wild animals

No wild animals involved in this study.

Reporting on sex

The current study emphasized the multimodal Alzheimer's diagnosis effects of DGC probe, which should not be significantly affected by sex. We used female mice mostly in this study for APP/PS1 model, which is easy to develop. Meanwhile, we supplemented in vivo assessments on both female and male mice, no obvious differences were observed. Therefore, sex is not a consideration in this study and these results could be applied to both sexes.

Field-collected samples

No field-collected samples involved in this study.

Ethics oversight

All animal procedures were performed under an approved protocol of the Ethics Committee of Beijing University of Technology, China.

Note that full information on the approval of the study protocol must also be provided in the manuscript.

Plants

Seed stocks

Report on the source of all seed stocks or other plant material used. If applicable, state the seed stock centre and catalogue number. If plant specimens were collected from the field, describe the collection location, date and sampling procedures.

Novel plant genotypes

Describe the methods by which all novel plant genotypes were produced. This includes those generated by transgenic approaches, gene editing, chemical/radiation-based mutagenesis and hybridization. For transgenic lines, describe the transformation method, the number of independent lines analyzed and the generation upon which experiments were performed. For gene-edited lines, describe the editor used, the endogenous sequence targeted for editing, the targeting guide RNA sequence (if applicable) and how the editor was applied.

Authentication

Describe any authentication procedures for each seed stock used or novel genotype generated. Describe any experiments used to assess the effect of a mutation and, where applicable, how potential secondary effects (e.g. second site T-DNA insertions, mosiacism, off-target gene editing) were examined.
